# Eutectic Itraconazole and Posaconazole System and the Advantages of Its Use in the Amorphous Form

**DOI:** 10.1021/acs.molpharmaceut.6c00458

**Published:** 2026-07-31

**Authors:** Justyna Knapik-Kowalczuk, Abu T. M. Serajuddin, Paulina Poloczek, Mariya Mathew, Julia Cichocka-Łokuciejewska, Marian Paluch

**Affiliations:** † Institute of Physics, Faculty of Science and Technology, 49568 University of Silesia in Katowice, 75 Pułku Piechoty 1A, 41-500 Chorzów, Poland; ‡ College of Pharmacy and Health Sciences, 4131 St. John’s University, Queens, New York 11432, United States; § Department of Physical Chemistry, Faculty of Pharmacy, Medical University of Gdańsk, Al. Gen. J. Hallera 107, 80-416 Gdańsk, Poland

**Keywords:** itraconazole, posaconazole, eutectic mixture, co-amorphous systems, physical stability, liquid crystalline ordering

## Abstract

Itraconazole (ITR) and posaconazole (POS) exhibit closely related molecular structures but differ significantly in their ability to form liquid crystalline (LC) phases. This makes them an attractive model system to investigate how subtle structural differences influence molecular organization in the amorphous state. In this work, we investigate coamorphous systems composed of ITR and POS prepared via melt-quenching, focusing on the interplay between molecular dynamics, physical stability, and LC ordering. Differential scanning calorimetry (DSC) and broadband dielectric spectroscopy (BDS) reveal that the glass transition temperature remains nearly unchanged over a wide compositional range, indicating comparable molecular mobility of all investigated systems. Despite this similarity, a pronounced, nonmonotonic dependence of crystallization kinetics on composition is observed, with a clear stability maximum at the eutectic ratio. Strikingly, this specific composition is also associated with the complete disappearance of the LC ordering characteristic of neat ITR. While ITR-rich mixtures close to the eutectic point still exhibit residual LC transitions, the eutectic composition yields a fully disordered amorphous phase. These results demonstrate, for the first time, that coamorphization at the eutectic composition might suppress LC ordering. The findings highlight the critical role of composition in governing both molecular organization and stability in amorphous pharmaceuticals.

## Graphical abstract

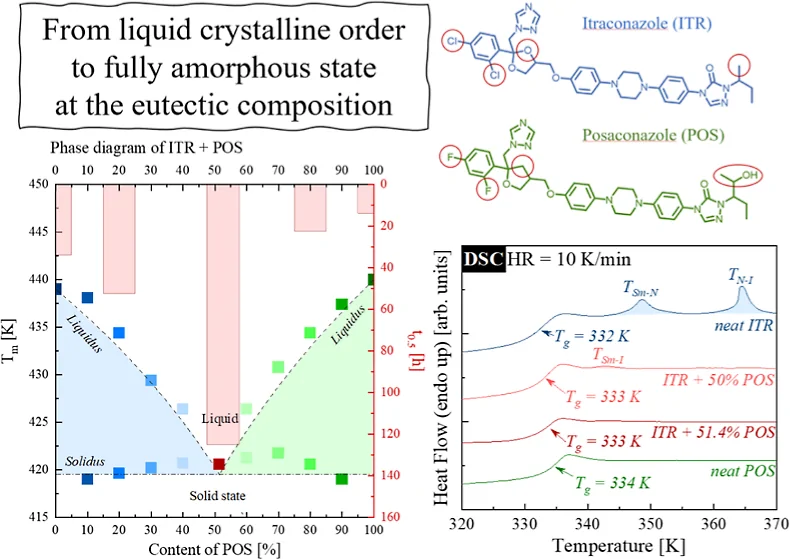

## 1 Introduction

The poor aqueous solubility of active pharmaceutical ingredients (APIs) remains a major challenge in contemporary drug development. This issue is particularly pronounced among antifungal triazoles, such as itraconazole (ITR) and posaconazole (POS), which, despite their high therapeutic efficacy, exhibit very limited bioavailability due to poor dissolution rates.^1,2^ Both APIs are classified as Biopharmaceutics Classification System II (BCS II) compounds,^3,4^ with aqueous solubility values at neutral pH of approximately 1–4 ng/mL for ITR and 200 ng/mL for POS.^5,6^

Both ITR and POS are structurally similar, rod-like molecules with the primary chemical difference between them being that POS is a tetrahydrofuran analog of ITR, in which the two chlorine atoms in the phenyl ring have been replaced by fluorine, and the triazolone side chain is hydrogenated (see Figure 1). In the solid state, they exhibit similar thermal properties; their melting points are similar, typically 438–445 K^7–9^ for ITR and 443–445 K for POS,^10,11^ and both exhibit nearly identical glass transition temperatures (*T*_*g ITR*_ = 332 K, and *T*_*g POS*_ = 334 K) immediately after amorphization.^9,13^ Despite these similarities, the two compounds differ fundamentally in their ability to form mesophases. When quench-cooled, ITR exhibits domain-like liquid crystalline (LC) ordering, characterized by smectic (*SmA*) and nematic (*N*) phases, while POS does not reveal such behavior.^13,14^

**Figure 1:**
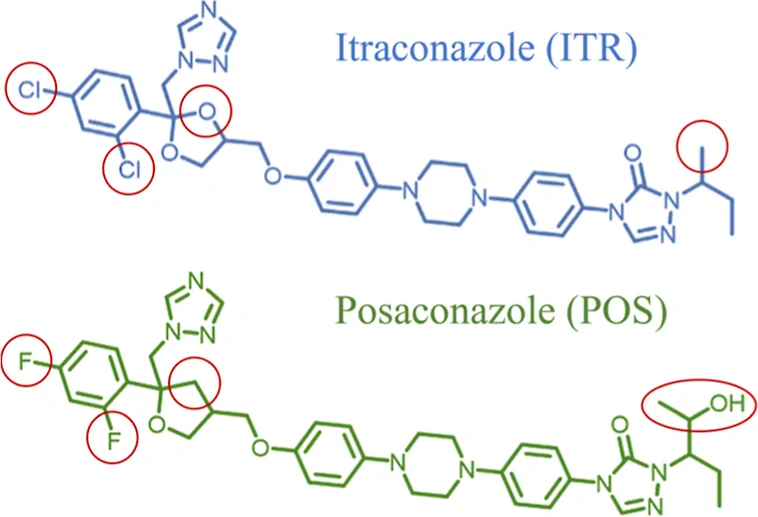
Chemical structures of ITR (top) and POS (bottom), highlighting the key structural differences between the two molecules.

The above-mentioned difference in liquid crystallinity between the two compounds impacts the physical stability of their amorphous forms. In general, the influence of LC ordering on amorphous physical stability cannot be considered straightforward. On the one hand, LC domains may act as structurally preorganized regions that facilitate molecular alignment during crystal growth. On the other hand, partial ordering within LC domains may locally restrict molecular mobility and increase the energetic barrier for nucleation, thereby slowing down crystallization. The latter scenario most likely applies to neat ITR, whose LC ordering has been associated with much higher physical stability compared to fully amorphous POS. According to the classification proposed by Baird et al., POS belongs to class II glass formers, characterized by limited stability above *T*_*g*_, whereas ITR, due to its LC ordering, is classified as a more stable class III system.^12^ Interestingly, although the partial ordering of ITR into LC domains contributes to its high physical stability, it also limits the solubility enhancement typically expected from amorphization. The presence of *SmA* and *N* domains in the melt-quenched material reduces the thermodynamic driving force for dissolution, as compared to a fully disordered amorphous phase.

Considering the aforementioned structural and physicochemical similarities and differences between ITR and POS, we hypothesized that their crystalline binary combination could form a eutectic system that might also exhibit improved performance in the amorphous state, namely, enhanced aqueous solubility and greater physical stability. Special attention was given to the potential suppression of LC ordering in ITR and stabilization of amorphous POS, known for its high recrystallization tendency.

In this work, we systematically investigated the thermal properties of both crystalline and amorphous forms of the ITR + POS binary system across the full compositional range using differential scanning calorimetry (DSC). Broadband dielectric spectroscopy (BDS) was employed to investigate the similarities and differences in dielectric response, including the temperature dependence of structural relaxation time (τ_α_(*T*)), the shape of the α-relaxation peak, the presence of δ-relaxation, and the tendency toward recrystallization among neat ITR, neat POS, and their selected binary coamorphous compositions. Finally, to confirm the observed stabilization effect and partially evaluate the sequence and nature of phase formation during crystallization of this coamorphous mixture, long-term physical stability studies were performed using powder X-ray diffraction (XRD). Our study identified the eutectic composition and revealed its unique ability to (i) eliminate mesophase formation of quench-cooled ITR and (ii) enhance physical stability of both quench-cooled parental components. Isothermal and nonisothermal experiments demonstrated that such a coamorphous composition suppresses recrystallization significantly, not only compared to neat components but also to other compositions of the mixture. These findings highlight the potential of coamorphous API + API combinations at eutectic concentration as a formulation strategy for poorly water-soluble crystalline drugs that are simultaneously highly unstable in the amorphous state.

## 2 Materials and Methods

### 2.1 Materials

Itraconazole (ITR, crystalline white powder, purity ≥98.5%, *M*_*w*_ = 705.64 g/mol) and posaconazole (POS, crystalline white powder, purity ≥95%, *M*_*w*_ = 700.79 g/mol) were purchased from Fisher Scientific International (Pittsburgh, PA, USA) and Chemshuttle (Burlingame, CA, USA), respectively.

### 2.2 Differential Scanning Calorimetry (DSC)

The thermal properties of neat, both crystalline and amorphous ITR and POS, as well as their binary mixtures, were investigated using Q200 DSC analyzer (TA Instruments, New Castle, DE, USA). For this purpose, powder samples of mass between 5 and 10 mg, either of a neat APIs or their binary physical mixtures, were placed into aluminum crucibles (40 μL). As a reference, an empty aluminum crucible was utilized. The experiments were performed at controlled nitrogen gas flow rates equal to 50 mL/min. Instrument calibration for temperature and enthalpy was performed using indium and zinc standards. The melting point of the neat compounds was determined from the onset of the melting endotherm. For the binary mixtures, the solidus and liquidus temperatures used for construction of the phase diagram were determined from the onset and the maximum of the melting endotherm, respectively. Glass transition temperature (*T*_*g*_) was identified as the midpoint of the heat capacity increment. All samples were measured with different heating rates. Each experiment was conducted at least in triplicate.

### 2.3 Broadband Dielectric Spectroscopy (BDS)

The molecular dynamics of neat amorphous ITR, POS, and their coamorphous mixtures containing 20, 51.4, and 80 wt % of POS were investigated using a Novo-Control GMBH Alpha dielectric spectrometer. Prior to the dielectric measurements, the crystalline samples were melted by means of the laboratory hot plate directly on the lower electrode of the dielectric capacitor. Subsequently, silica spacer fibers were placed on the molten sample, and the upper electrode was placed on top. The entire capacitor was then rapidly transferred onto a copper plate to obtain the amorphous state by fast quenching. The vitrified samples were then mounted in the dielectric spectrometer. Dielectric measurements were carried out over a wide frequency range from 10^–1^ Hz to 10^6^ Hz. For nonisothermal experiments, the samples were heated in 2 K steps within the temperature ranges of 329–403 K for ITR, 331–383 K for POS, and 329–393 K for the mixtures. Temperature control was achieved using a Quattro temperature controller, ensuring stability better than 0.1 K. For isothermal crystallization experiments, the vitrified samples were heated directly from room temperature to the selected crystallization temperature and maintained under isothermal conditions throughout the dielectric measurements. The examined systems were measured in a parallel-plate cell made of stainless steel (diameter of 15 mm, and a 0.1 mm gap provided by silica spacer fibers).

### 2.4 X-Ray Diffraction (XRD)

PXRD patterns of ITR, POS, and their physical or coamorphous mixtures were recorded using a Shimadzu XRD-6000 diffractometer (Shimadzu, Kyoto, Japan) equipped with a Ni filter and monochromatic Cu-Kα radiation source. The diffractometer was operated at 40 kV and 30 mA with a copper anode tube, and the scanning was performed from 5° to 40° of 2θ at 2° per min.

## 3 Results and Discussion

### 3.1 Thermal Properties of the Crystalline ITR + POS Systems

To gain insight into the thermal behavior of the crystalline neat ITR, neat POS, and their binary physical mixtures, differential scanning calorimetry (DSC) was employed. The DSC scans were obtained during heating at a rate of 5 K/min, and results are summarized in Figure 2a, where the neat ITR exhibited a single sharp endothermic event at 439 K, corresponding to its melting, while the neat POS exhibited two endothermic events: a minor transition at a lower temperature (∼408 K), followed by the main melting peak at 443 K. Such thermal behavior has been consistently reported for crystalline POS in the literature.^13^ While earlier studies associated the lower-temperature event with either a nematic-like transition or the melting of a metastable polymorphic form,^14,15^ more recent investigations combining DSC with variable-temperature PXRD demonstrated that it originates from a reversible solid-state polymorphic transformation from Form I to Form I′ occurring prior to melting.^16^ Importantly, identical DSC behavior was observed for commercially available POS samples obtained from four independent suppliers in our laboratory, confirming that this additional transition is a reproducible feature of crystalline POS rather than evidence of sample impurity. These thermal characteristics were used as a reference to identify the impact of particular APIs on their thermal properties in the crystalline mixtures as well as to establish the ability to form an eutectic composition.

**Figure 2:**
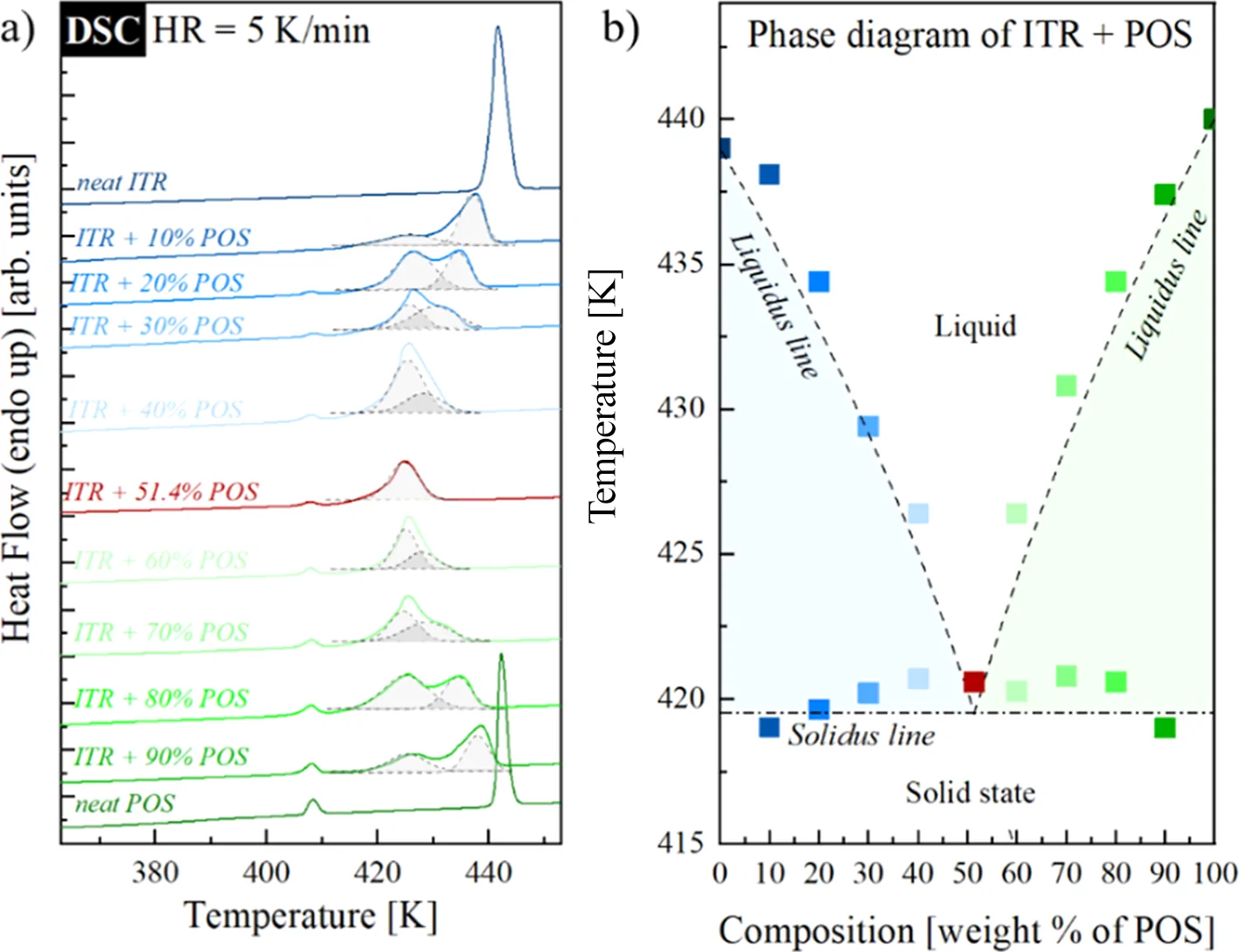
(A) DSC thermograms (heating rate: 5 K/min) of ITR + POS crystalline mixtures with increasing POS content. (b) Binary phase diagram of the ITR + POS system constructed based on experimental data and theoretical approach. Data points correspond to melting temperatures determined experimentally, while dashed lines represent phase boundaries calculated using the Schröder–van Laar equation. The eutectic point is located at ITR + 51.4 wt % POS and 420.7 K.

Upon mixing ITR and POS in varying proportions, the DSC thermograms of the resulting crystalline binary mixtures revealed composition-dependent melting behavior. As illustrated in Figure 2a, three endothermic events could be distinguished for most compositions: (i) a minor low–temperature transition observed below 410 K, (ii) a eutectic-related melting peak with an onset at 420 K, and (iii) a high-temperature endotherm corresponding to the melting of the excess component (either ITR or POS). The presence and relative intensity of these thermal events varied with composition.

As discussed earlier, the first low-temperature event, also present in neat POS, has been attributed either to the melting of metastable a polymorph or to the residual impurities in the POS sample (the interpretation depending on the literature). Consistent with the behavior of neat POS discussed above, this low-temperature event can be assigned to the reversible Form I → Form I′ solid-state transformation of POS. In the present study, this transition was consistently observed in all POS-containing mixtures, while its onset temperature remained essentially independent of the ITR content. This composition-independent behavior supports its assignment to an intrinsic polymorphic transformation of POS and indicates that the event is unrelated to eutectic melting or to interactions specific to a particular mixture composition.

The second and third endothermic events, which are, respectively, associated with eutectic melting and melting of the excess component, showed the expected evolution for a eutectic-forming system. As the composition approached 51.4 wt % POS, these two peaks gradually converged. In this composition, a single, sharp melting endotherm was observed at 420 K. The absence of any additional melting signals confirms the formation of a eutectic mixture, in which both components simultaneously melt as a single phase.

Based on DSC data, the phase diagram of the ITR + POS system was constructed and is presented in Figure 2b. As can be seen, the phase diagram displays a typical V-shaped curve with clearly identified solidus and liquidus lines, and a eutectic point located at ITR + 51.4 wt % POS. The experimentally determined phase diagram shows good agreement with the theoretical prediction based on the Schröder van Laar model (see dashed lines), which enables the calculation of liquidus temperatures using the melting points (*T*_*m*_) and enthalpies of fusion (Δ*H*_*f*_) of the pure components, according to the following equation^17^

1 $$T={\left[\frac{1}{T_{m}}-\frac{R\ln\left(\chi \right)}{\Delta H_{f}}\right]}^{-1}$$

where χ represents the molar fraction of the given substance in the system, while *R* represents the gas constant. Since the Schröder van Laar model is commonly applied to estimate the shape of liquidus lines in binary systems under the assumption of ideal mixing in the liquid phase, the agreement with experimental data supports the conclusion that the ITR + POS system exhibits nearly ideal mixing behavior in the molten state.

Having established the eutectic composition and melting behavior of the crystalline ITR + POS system, attention was next turned to its performance in the amorphous state, with particular focus on the glass transition, thermal stability, and the presence of liquid crystalline domains.

### 3.2 Thermal Properties of the Amorphous ITR + POS Systems

To assess the thermal properties of the amorphous ITR + POS systems, a series of melt-quenched samples with varying compositions was investigated by DSC using a standard heating rate of 10 K/min. The obtained DSC thermograms are presented in Figure 3a.

**Figure 3:**
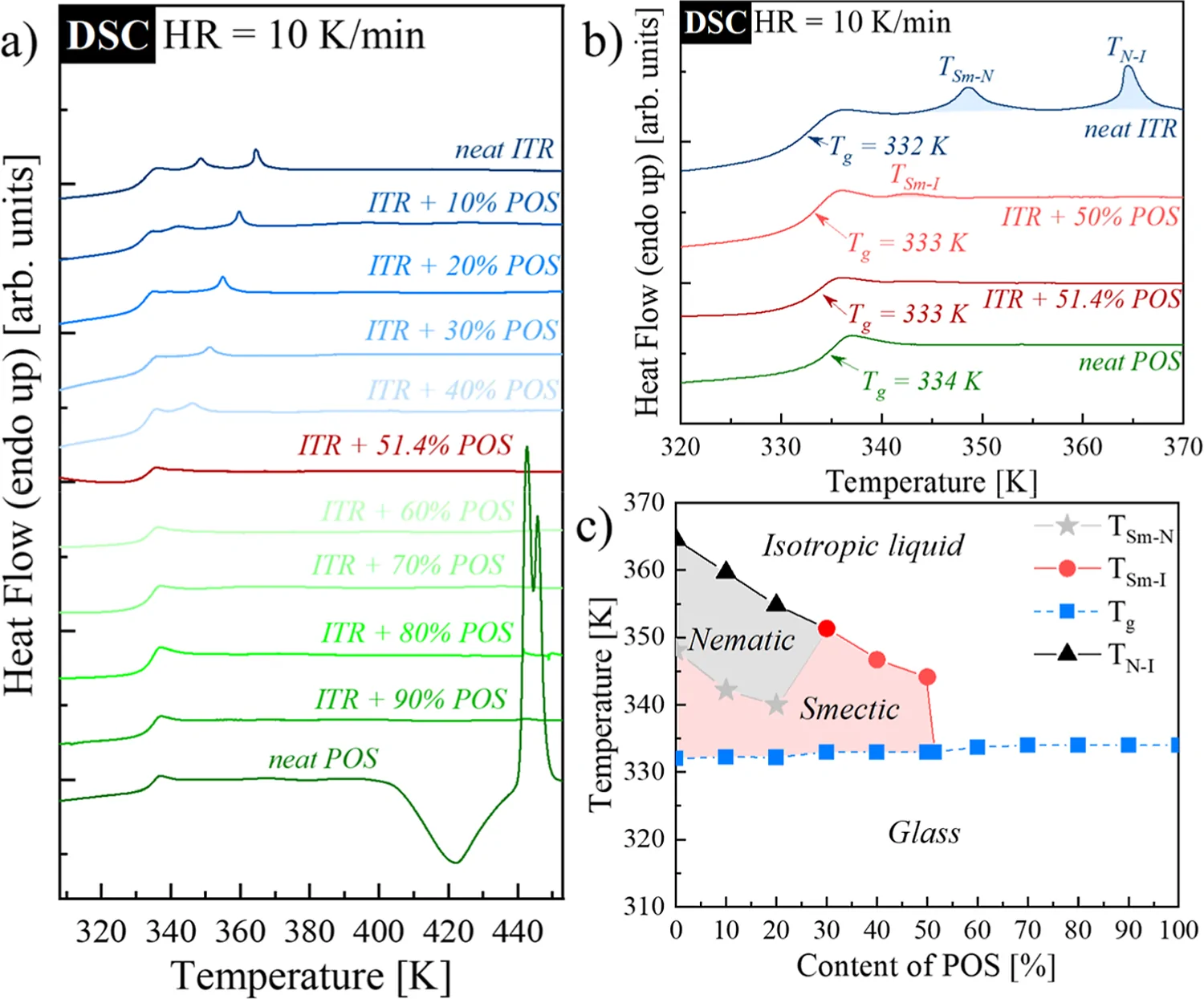
(A) DSC heating thermograms (10 K/min) of neat amorphous ITR, neat amorphous POS, and their coamorphous mixtures across the full compositional range. (b) Close-up of the low-temperature region highlighting glass transitions and mesophase behavior in selected compositions. (c) Temperature–composition diagram summarizing the observed transitions. The eutectic composition (ITR + 51.4 wt % POS) marks the boundary beyond which mesophase transitions are fully suppressed, and only glass transition remains detectable.

All investigated compositions exhibited a single glass transition event (*T*_*g*_), with values ranging between 332 and 334 K. Following the glass transition, additional endothermic events were observed in several mixtures, corresponding to the presence of liquid crystalline mesophases. In the case of neat ITR as well as for mixtures containing 10 and 20 wt % of POS, two distinct endothermic events were detected: the smectic-to-nematic transition (*T*_*Sm–N*_) and the nematic-to-isotropic transition (*T*_*N–I*_). For neat ITR, these transitions occurred at T = 348 K and T = 365 K, respectively. In the 10 and 20 wt % of POS mixtures, both transitions remained detectable, but their onset temperatures were shifted toward lower values, indicating a destabilization of the LC ordering with increasing POS content. Interestingly, in the intermediate compositions ranging from 30 to 50 wt % of POS, only a single endothermic event was observed in the supercooled liquid region. This transition is more likely due to a direct transformation from a *SmA* to the isotropic liquid, bypassing the nematic state. This observation suggests that the *N* phase is suppressed before the *SmA* ordering is fully destabilized, simplifying the phase sequence as a result of molecular disruption induced by POS. The same pattern of behavior has been reported for ITR-based systems modified with small-molecular additives such as glycerol, where increasing the additive concentration led to the disappearance of the *N* phase and a direct smectic-to-isotropic transition.^18^ Upon further increasing the amount of POS in ITR + POS mixtures, no LC-related transitions were observed in the DSC thermograms. Interestingly, the concentration at which LC ordering is no longer detectable coincides exactly with the eutectic composition (i.e., 51.4 wt % of POS). To better illustrate this finding, a magnified view of the LC transition region is shown in Figure 3b, where thermograms of neat ITR, neat POS, and two selected mixtures–one with 50 wt % of POS and the other corresponding to the eutectic composition–are presented. While the 50 wt % POS mixture still displays a residual smectic-to-isotropic transition, it disappears completely in the eutectic mixture (51.4 wt % POS). To our knowledge, no previous reports have demonstrated a correlation between eutectic composition and the suppression of LC ordering in binary pharmaceutical mixtures. On the one hand, this observation may point to a previously unrecognized relationship between the eutectic composition and the suppression of LC ordering in one of the mixture’s components. However, this effect could be coincidental and specific to the ITR + POS system. To test whether the observed trend is universal or specific to the ITR + POS system, it would be worth conducting analogous studies in the future on API + API combinations that reveal liquid–crystalline properties.

To provide a comprehensive overview of the thermal behavior described above across the full compositional range of the coamorphous ITR + POS system, Figure 3c presents a phase diagram summarizing the temperatures of the observed thermal events, i.e., the glass transition temperature (*T*_*g*_), smectic-to-nematic transition temperature (*T*_*Sm‑N*_), and nematic-to-isotropic transition temperature (*T*_*N‑I*_), as a function of POS content. The phase diagram was constructed using *T*_*g*_ values determined as the midpoint of the heat-capacity increment, whereas the temperatures of the mesophase transitions were taken as the maxima of the corresponding endothermic peaks observed in the DSC thermograms. The diagram clearly visualizes the narrowing temperature window for LC mesophases and their abrupt disappearance in the eutectic region.

Beyond the suppression of LC ordering of ITR in the presence POS, the DSC thermograms also provided preliminary insight into the physical stability of the coamorphous ITR + POS mixtures. As can be noted, none of the studied compositions exhibited recrystallization upon heating (with a rate of 10 K/min), even those containing high amounts of POS, which is known to crystallize readily. This finding indicates that ITR effectively stabilizes amorphous POS even if it does not significantly impact its *T*_*g*_.

### 3.3 Dielectric Insights into ITR + POS Supercooled Mixtures

The small difference in glass transition temperatures observed for neat ITR, neat POS and their mixtures suggests that their molecular mobilities are not substantially different. This is particularly intriguing considering the calorimetric findings, which indicate that amorphous ITR can effectively stabilize amorphous POS, despite having little effect on its molecular dynamics–a factor often regarded as a key determinant of physical stability in amorphous materials. The main objective of this section is to examine the similarities and differences in dielectric response–which provides insight into molecular dynamics–among neat ITR, neat POS, and their selected binary coamorphous compositions to better understand the observed stabilization effect.

Before analyzing the effect of mixing on dielectric response, it is essential to first recall the dielectric behavior of the neat components–ITR and POS–in the supercooled liquid state (*T* > *T*_*g*_). Figure 4 presents dielectric loss spectra (ε’’) as a function of frequency for neat ITR (panel a) and neat POS (panel b), collected at temperatures from 329 to 403 K and from 331 to 383 K for ITR and POS, respectively.

**Figure 4:**
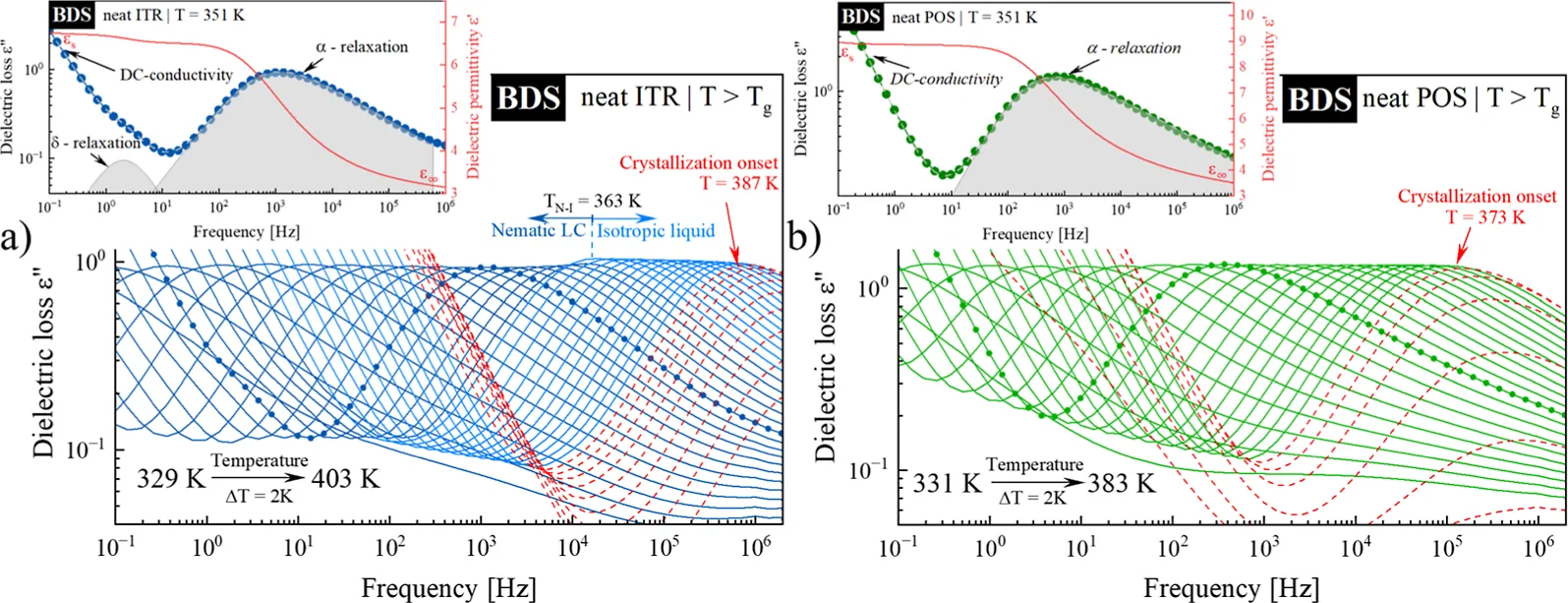
Data obtained utilizing broadband dielectric spectroscopy (BDS) data for neat amorphous itraconazole (a) and posaconazole (b) measured upon heating above *T*_*g*_. The main panels present the evolution of dielectric loss spectra (ε″) in the frequency domain as a function of temperature (Δ*T* = 2 K). For ITR (a), characteristic transitions associated with mesophase formation are observed, including the nematic-to-isotropic transformation, followed by crystallization onset at 387 K. In contrast, POS (b) displays no mesophase transitions and crystallizes earlier, with an onset at 373 K. Insets show selected–representative–dielectric spectra collected at 351 K, highlighting contributions from α-relaxation, DC-conductivity, and δ-relaxation processes.

As shown in Figure 4a, the dielectric loss spectra of supercooled ITR reveal the presence of two distinct relaxation processes: slower δ-relaxation and a faster α-relaxation. According to previous dielectric studies on itraconazole, the δ-process originates from flip–flop rotations of the molecules about their short molecular axis, whereas the α-process corresponds to the cooperative structural relaxation associated with precessional (tumbling) motions around the long molecular axis.^19^ The simultaneous presence of these two relaxation processes is a well-established dielectric hallmark of orientationally ordered LC domains of the neat supercooled ITR. For improved clarity, the inset in panel a present the real (ε′) and imaginary (ε′′) parts of the complex permittivity spectrum measured at 351 K (marked in the main plot as the line with scatter). The spectrum can be accurately described by the superposition of the α- and δ-relaxation peaks along with DC conductivity, with each contribution individually fitted using the Havriliak-Negami (HN) fit function.^15^ With increasing temperature, the position of both relaxation peaks shifts toward higher frequencies, reflecting the acceleration of molecular motions. At *T*_*N‑I*_ = 363 K, the characteristic signature of the nematic-to-isotropic phase transition can be identified: the δ-relaxation vanishes. In contrast, the dielectric spectra of neat POS (Figure 4b) reveal only a single relaxation process (α-relaxation) without any additional slower contribution. This mode also shifts toward higher frequencies with increasing temperature, indicating the acceleration of POS’s molecular dynamics. It is worth pointing out that the described behavior, i.e., lack of δ-relaxation in POS’s dielectric response, is consistent with the absence of LC mesophases in this material, as also demonstrated in the DSC measurements. Furthermore, during further heating of both investigated samples, the recrystallization process was clearly captured. The crystallization onset temperatures were identified as 387 K for ITR and 373 K for POS, based on a pronounced drop in the intensity of the α-relaxation peak. Dielectric spectroscopy is a powerful tool for monitoring recrystallization phenomena because it is sensitive to the number of dynamically reorienting dipoles (Δε ∝ N·μ^2^), which decreases as the system crystallizes.^20^ This ability will be particularly leveraged in the next section, where time-dependent isothermal dielectric studies will be conducted to investigate the crystallization kinetics of the studied samples. Before that, however, we turn our attention to the dielectric response of the coamorphous ITR + POS systems.

Panels a to c in Figure 5 present dielectric loss spectra measured above *T*_*g*_ for three representative compositions of the coamorphous ITR + POS mixtures: one rich in POS, one rich in ITR, and one corresponding to the eutectic composition. The data reveal three important features. First, all samples exhibit a well-defined α-relaxation peak whose position shifts toward higher frequencies with increasing temperature, accompanied by a characteristic DC conductivity contribution. Second, the spectra of the ITR + 20 wt % of POS sample exhibit a clearly detectable but reduced in comparison to neat ITR δ-relaxation process, indicating the persistence of LC ordering in this composition; notably, the *T*_*N‑I*_ can be determined as 353 K. Third, only the sample with the highest POS content (i.e., ITR + 80 wt % of POS) displayed signs of recrystallization during the measurement. The remaining panels of Figure 5 provide a comparative overview of dielectric behavior across the series (including neat components), emphasizing the similarities and differences in dielectric response among the systems studied.

**Figure 5:**
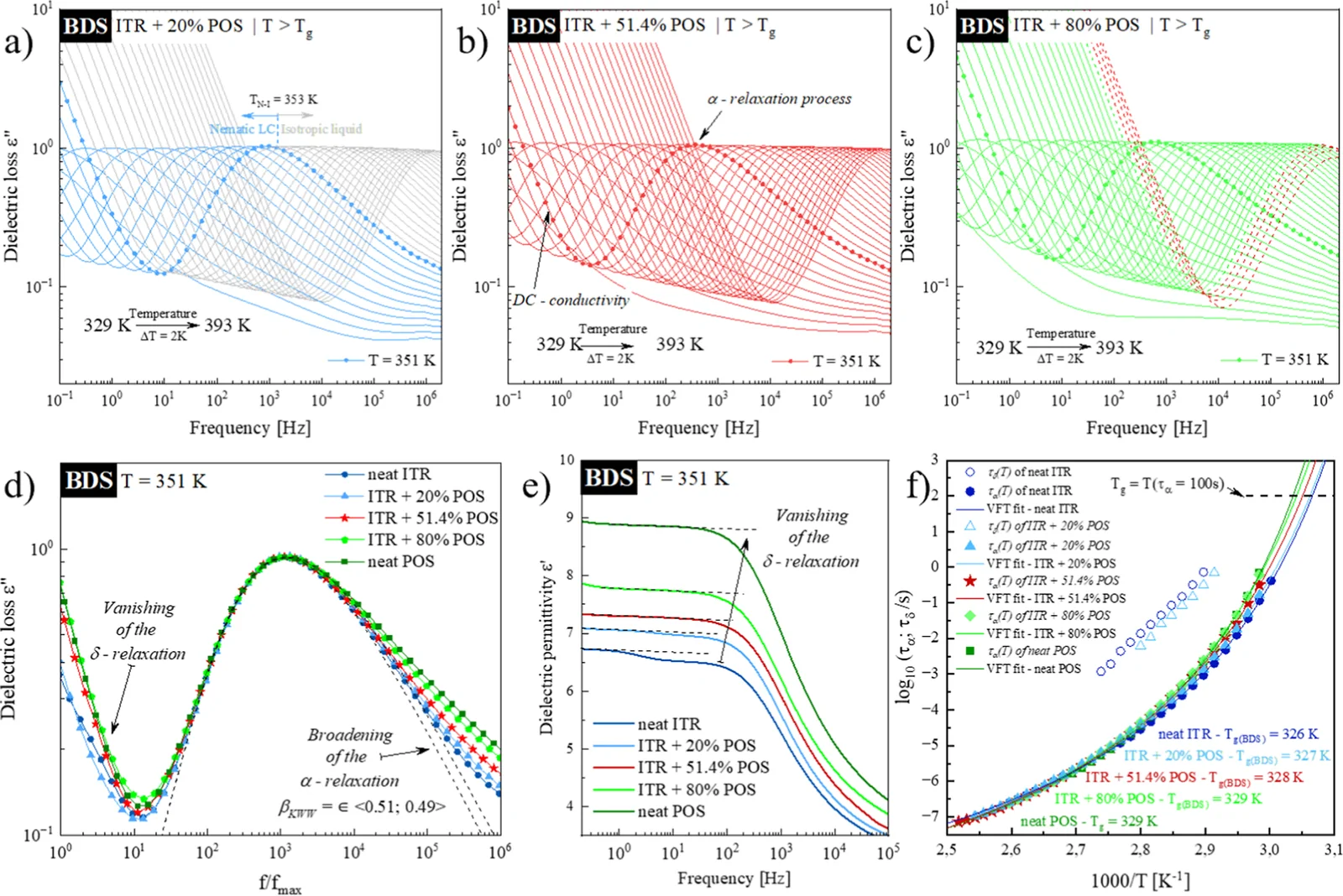
BDS results for ITR + POS mixtures with 20%, 51.4% (eutectic), and 80% POS content. Panels (a–c) show dielectric loss spectra (ε″) collected at various temperature conditions upon heating above T_g_ (Δ*T* = 2 K) for selected compositions. The α-relaxation process, DC-conductivity, and LC transitions are highlighted. (d) Comparison of the dielectric loss spectra (ε″) collected at 351 K, indicating systematic broadening of the α-relaxation peak and the vanishing of the δ-relaxation with increasing POS content. (e) Comparison of dielectric permittivity spectra (ε′) collected at 351 K illustrating the disappearance of the δ-relaxation. (f) Temperature dependence of α-relaxation times of all investigated samples fitted using the VFT equation.

Panel d of Figure 5 compares the shape of the dielectric loss spectra for all measured samples at a representative temperature above *T*_*g*_ (*T* = 351 K). From the high-to low-frequency range, two key observations can be made. First, the shape of the α-relaxation peak shows a subtle broadening with increasing POS content. The width parameter (β_*KWW*_) ranges from 0.51 for neat ITR to 0.49 for neat POS, suggesting comparable degrees of dynamic heterogeneity among the samples. Second, the progressive disappearance of the δ-relaxation reflects the compositional suppression of LC alignment. This suppression is even more evident in panel e, where the real part of the complex permittivity is presented, as the complex permittivity is not affected by DC conductivity. In this representation, the δ-relaxation manifests as a characteristic step in ε′(*f*) at low frequencies. As POS content increases, this stepwise feature gradually diminishes and ultimately disappears, confirming the disruption of orientational order within the mixtures.

Finally, Figure 5f summarizes the temperature dependencies of the relaxation times for both α- and δ-processes across all systems studied. To determine τ_α_ and τ_δ_, the dielectric spectra were fitted using one or two (depending on composition) HN functions along with a DC conductivity term. The general form of the single HN function with conductivity correction is given by^21^

2 $$\varepsilon_{HN}^{*}\left(\omega \right)=\varepsilon^{\prime }\left(\omega \right)-i\varepsilon^{\prime \prime }\left(\omega \right)=\varepsilon_{\infty }+\frac{\Delta \varepsilon }{{\left[1+{\left(i\omega \tau_{HN}\right)}^{a}\right]}^{b}}+\frac{\sigma_{DC}}{\varepsilon_{0}i\omega }$$

where ε_∞_ is the high-frequency permittivity limit, Δε is the dielectric strength, ω = *2*π*f*, τ_*HN*_ is the HN relaxation time, and *a* and *b* describe symmetric and asymmetric broadening. The relaxation times τ_α_ and τ_δ_ were subsequently calculated from τ_*HN*_ using the following transformation

3 $$\tau_{\alpha /\delta }=\tau_{HN}{\left[\sin\left(\frac{\pi a}{2+2b}\right)\right]}^{-1/a}{\left[\sin\left(\frac{\pi ab}{2+2b}\right)\right]}^{1/a}$$

As shown, the temperature evolution of the δ-relaxation times for neat ITR in the investigated temperature window follows Arrhenius behavior. In order to describe the temperature evolution of τ_α_ in the supercooled liquid region for all studied systems, the Vogel–Fulcher–Tammann (VFT) equation was employed:

4 $$\tau_{\alpha }\left(T\right)=\tau_{\infty }\exp\left(\frac{B}{T-T_{0}}\right)$$

where τ_∞_, *B*, and *T*_*0*_ are fitting parameters,^21–24^ Here, τ_∞_ represents the preexponential factor related to the vibrational time scale (typically between 10^–11^ and 10^–14^ s), *T*_*0*_ is the Vogel temperature corresponding to the divergence of relaxation time, and *B* = *DT*_*0*_, with *D* being the parameter that quantifies the deviation from simple Arrhenius behavior.

The VFT fits are shown as solid lines in Figure 5f, while the fitting parameters are summarized in Table 1. These fits were further used to estimate the kinetic glass transition temperature (*T*_*g*_) for each composition using the standard criterion of τ_α_(*T*_*g*_) = 100 s. As indicated, the *T*_*g*_ values obtained from BDS increase systematically with rising POS content, closely mirroring the trend observed in DSC measurements. It should be noted that the *T*_*g*_ values obtained by the two methods differ slightly. The observed discrepancy between the kinetic and calorimetric *T*_*g*_ values is mainly due to the different heating rates used in these experiments.

**Table 1:** Comparison of the Values of *T*_*g*_ Obtained From DSC and BDS, and VFT Fitting Parameters for Neat Amorphous ITR, Neat Amorphous POS, and Their Binary Amorphous Mixtures Having 20, 51.4 and 80 wt % of POS

sample	log τ_∞_ [s]	*B* = *DT*_*0*_ [K]	*T*_*0*_ [K]	*T*_*g BDS*_ [K]	*T*_*g DSC*_ [K]
neat ITR	–10.7 ± 0.1	842 ± 24	297.6 ± 0.7	326	332
ITR + 20% POS	–10.8 ± 0.2	895 ± 32	296.4 ± 0.9	327	332
ITR + 51.4% POS	–11.4 ± 0.1	990 ± 15	295.8 ± 0.4	328	333
ITR + 80% POS	–11.5 ± 0.0	1001 ± 7	296.4 ± 0.2	329	334
neat POS	–11.2 ± 0.0	888 ± 6	300.0 ± 0.2	329	334

In summary, the presented dielectric data suggest that the observed improvement in the physical stability of amorphous POS upon the addition of ITR cannot be explained solely by the emergence of LC ordering or changes in molecular dynamics (e.g., antiplasticization). Although LC features are clearly evident in neat ITR, this sample exhibits a greater tendency to recrystallize than the less-ordered ITR + 20 wt % of POS mixture. Moreover, the eutectic composition–which shows no signs of LC alignment, as confirmed by both DSC and BDS measurements–also demonstrates excellent stability. These findings indicate that there is no direct relationship between LC ordering or molecular dynamics suppression and physical stability in ITR + POS systems, suggesting that other factors, possibly related to the specific intermolecular interactions associated with eutectic formation, may play here a crucial role. To further explore this issue, the next part of this study focuses on evaluating the physical stability of the ITR + POS quench-cooled systems under isothermal conditions, both above and below the *T*_*g*_.

### 3.4 Physical Stability Studies of Supercooled ITR + POS Systems

To determine how the recrystallization tendency of supercooled ITR + POS mixtures vary with composition; a series of time-dependent isothermal studies was performed using BDS. These experiments were designed not only to evaluate the impact of ITR on the suppression the recrystallization time of supercooled POS stored at stressed–elevated–temperature conditions but also to clarify the stabilizing mechanism, which could not be solely explained by the presence of LC ordering or antiplasticization effects, as discussed in the previous section. Many studies have reported enhanced physical stability of coamorphous systems at the eutectic composition compared to both the neat parent components and noneutectic mixtures.^25–28^ This behavior has been attributed to the strongest specific intermolecular interactions inherent to the eutectic composition. At the same time, recent studies have shown that this relationship is not universal and may depend on the specific binary system.^29,30^ Therefore, the nonisothermal results presented and discussed in Section: *Thermal Properties of the Amorphous ITR + POS Systems* and *Dielectric Insights into ITR + POS Supercooled Mixtures*, do not allow us to conclude whether the ITR + POS follows this commonly reported stabilization trend. To clarify this issue, three mixtures examined in the previous section–namely ITR + 20 wt % POS, the eutectic formulation (ITR + 51.4 wt % POS), and ITR + 80 wt % POS–were selected for time-dependent isothermal dielectric experiments, along with neat ITR and POS. During these measurements, the samples were stored at *T* = 363 K, and the spectra of the complex dielectric permittivity were recorded at a specific time intervals of 600 s, until complete recrystallization was observed. This specific temperature was selected based on several considerations. First, it provides sufficiently fast recrystallization kinetics for the neat amorphous components, allowing the entire crystallization process to be monitored within a single dielectric experiment. At the same time, 363 K remains sufficiently below the temperature at which crystallization becomes apparent during the nonisothermal BDS measurements presented in the previous section, enabling reliable determination of the induction time preceding recrystallization. Furthermore, for neat ITR, this temperature corresponds to the nematic-to-isotropic transition temperature (*T*_*N–I*_), ensuring that all investigated samples were measured entirely in the isotropic liquid state, without contributions from liquid-crystalline ordering.

Figure 6a shows representative ε′(*f*) spectra of neat POS collected over time. As shown, the dielectric permittivity decreases gradually with increasing storage time. This behavior reflects the progressive crystallization of the sample and can be attributed to a reduction in the number of dynamically reorienting dipoles within the system, which directly translates into a decrease in dielectric strength (Δε ∝ *N·*μ^2^) as discussed in the previous section.

**Figure 6:**
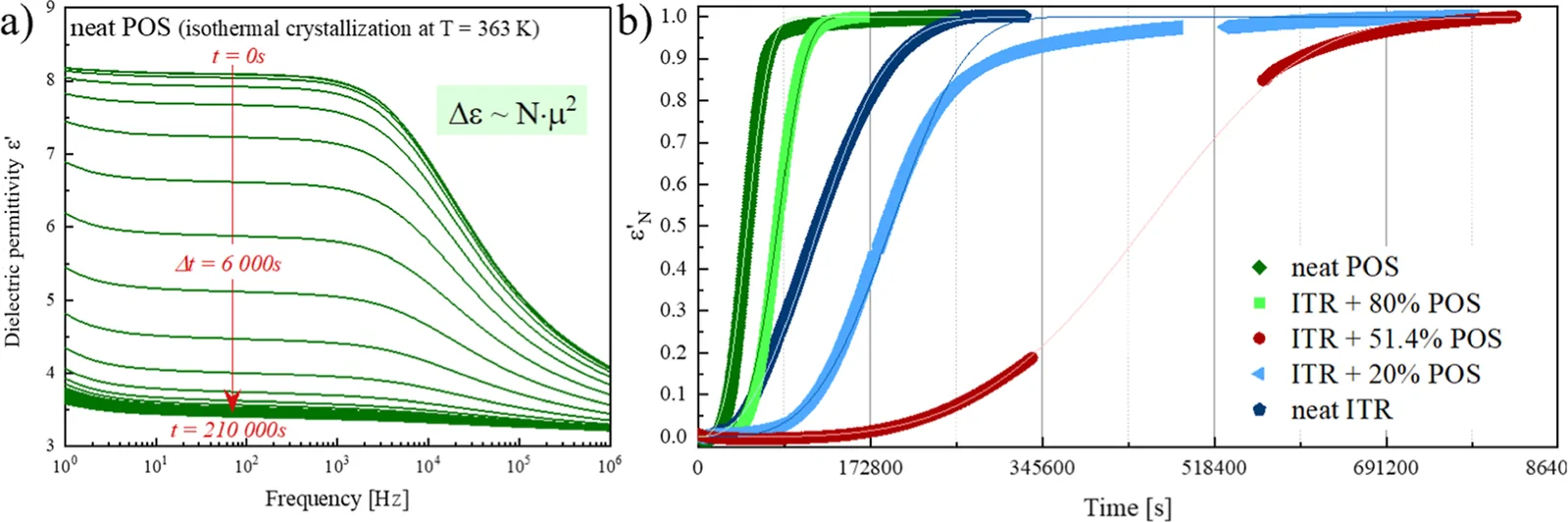
(A) Evolution of dielectric permittivity spectra (ε′) recorded for neat amorphous POS during isothermal crystallization at 363 K. The gradual decrease in dielectric strength over time reflects a reduction in the number of dipolar reorienting units, associated with progressive crystallization. (b) Normalized dielectric strength (ε′_n_) plotted as a function of time for selected ITR + POS compositions and neat APIs, illustrating the impact of composition on crystallization kinetics. The eutectic mixture (ITR + 51.4 wt % POS, red curve) exhibits the longest induction time and slowest crystallization rate, indicating significantly enhanced physical stability relative to both neat components and noneutectic mixtures.

The results of analogous measurements performed for the rest of the samples were normalized according to the following equation^31^

5 $$\varepsilon {’}_{N}\left(t\right)=\frac{\varepsilon ’\left(0\right)-\varepsilon ’\left(t\right)}{\varepsilon ’\left(0\right)-\varepsilon ’\left(\infty \right)}$$

where ε′(*0*) and ε′(∞) correspond to the initial and final permittivity values, respectively, and ε′(*t*) denotes the permittivity recorded at a given time. This normalization facilitates a direct comparison of crystallization kinetics across samples having different ε_*s*_ values. The normalized crystallization curves are collectively presented in Figure 6b. As shown, the samples investigated differ significantly in their physical stability under isothermal conditions. By comparing the crystallization half-life (*t*_*0.5*_) of all investigated materials–namely, neat POS (*t*_*0.5*_ ≈ 13.8 h), ITR + 80 wt % POS (*t*_*0.5*_ ≈ 22.5 h), ITR + 51.4 wt % POS (*t*_*0.5*_ ≈ 125.0 h), ITR + 20 wt % POS (*t*_*0.5*_ ≈ 52.5 h), and neat ITR (*t*_*0.5*_ ≈ 33.9 h)–one can clearly observe the highest physical stability for the amorphous ITR + POS system at the eutectic composition. This result supports the assumption previously discussed, that the recrystallization tendency of the coamorphous ITR + POS system follows the behavior expected for eutectic compositions. To enable clearer visualization and consequently, more accurate comparison of the physical stability among the investigated samples, the crystallization half-life times were plotted on the phase diagram shown in Figure 7.

**Figure 7:**
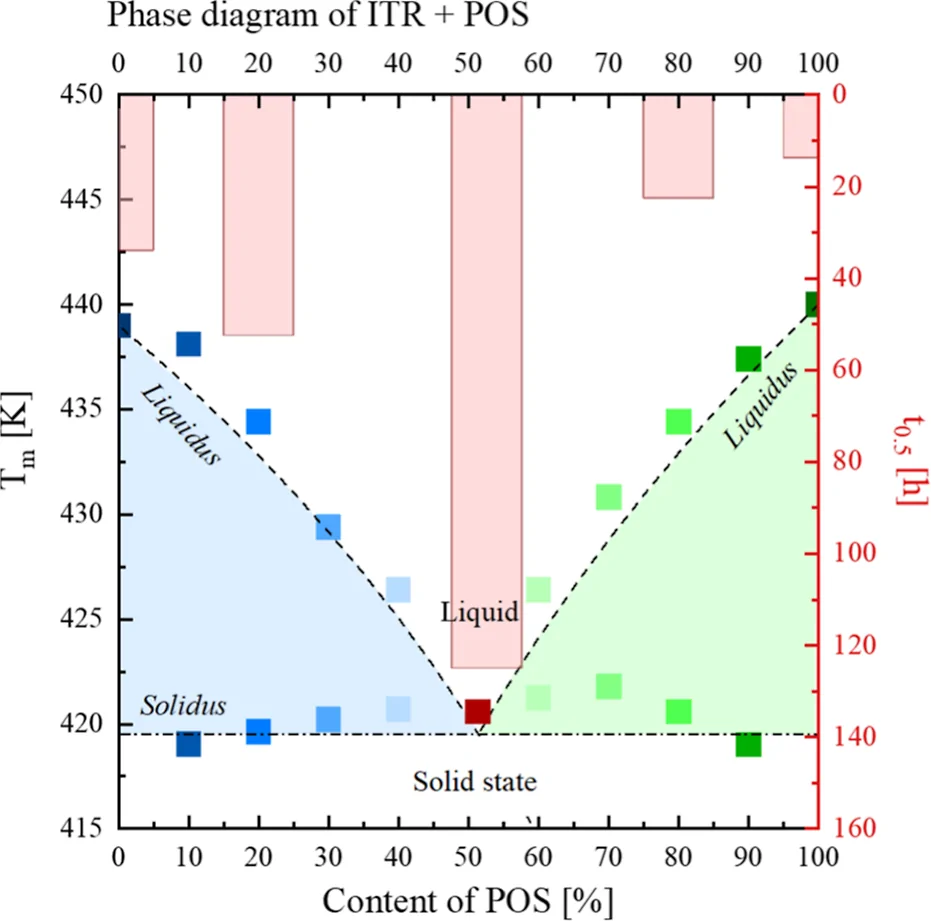
Phase diagram of the ITR + POS binary system, constructed based on melting temperatures obtained from DSC data (left axis). Superimposed red bars correspond to crystallization half-life times (*t*_*0.5*_) determined for selected compositions (right axis).

To quantitatively describe the crystallization behavior of the investigated samples their kinetic curves were fitted using the Avrami model. This model is one of the most widely used approaches for evaluating crystallization kinetics at a fixed temperature and is expressed by the following equation^20^

6 $$\varepsilon {’}_{N}\left(t\right)=1-\exp\left(-kt^{n}\right)$$

where *k* is the crystallization rate constant, and *n* is the Avrami exponent related to the nucleation and crystal growth mechanism. Table 2 summarizes the calculated values of the Avrami exponent, the crystallization rate constant, as well as the onset time, half-crystallization time, and the time to full recrystallization for all investigated samples. It is worth noting that, in addition to the overall increase in crystallization time as the eutectic composition is approached, a gradual rise in the *n* values is also observed. Although the Avrami exponent alone does not allow unambiguous identification of the crystallization mechanism, this observation suggests that crystallization of the coamorphous system at the eutectic composition may proceed through a more complex pathway compared to the remaining formulations. Such behavior may reflect differences in the relative contributions of nucleation and crystal growth or in the effective growth dimensionality. Importantly, a more complex crystallization pathway may also be associated with slower crystallization kinetics and, consequently, enhanced physical stability of the coamorphous system. This possibility is further examined in the following section using isothermal dielectric measurements performed under accelerated storage conditions.

**Table 2:** Avrami Parameters (k and n) Determined From Fits to Isothermal Dielectric Crystallization Data Collected at 363 K for Neat Amorphous ITR, POS, and Their Co-amorphous Mixtures^a^

sample	*k* [s^–n^]	*n*	*t*_*cryst.(onset)*_ [min]	*t*_*0.5*_ [min]	*t*_*cryst.(full)*_ [min]
neat ITR	(1.370 ± 0.002) × 10^–13^	2.50 ± 0.05	410 min	2036 min	4880 min
ITR + 20%POS	(1.910 ± 0.010) × 10^–20^	3.70 ± 0.10	934 min	3148 min	11390 min
ITR + 51.4%POS	(1.710 ± 0.003) × 10^–23^	4.00 ± 0.05	1588 min	7499 min	13746 min
ITR + 80%POS	(1.660 ± 0.020) × 10^–20^	3.90 ± 0.01	508 min	1349 min	2622 min
neat POS	(6.730 ± 0.040) × 10^–16^	3.20 ± 0.01	149 min	825 min	3440 min

a The table also includes the corresponding crystallization onset (*t*_*cryst (onset)*_), half-crystallization time (*t*_*0*.*5*_), and total crystallization time (*t*_*cryst.(full)*_).

To complement the crystallization studies performed by BDS for neat POS, neat ITR, and their coamorphous mixtures, powder X-ray diffraction (PXRD) studies were conducted under identical thermal conditions. These measurements were performed for the neat components and for the coamorphous mixture with eutectic composition (ITR + 51.4 wt % POS). The aim of the PXRD studies was 2-fold: first, to verify whether the eutectic coamorphous system, which was previously identified as the most physically stable composition, indeed maintained superior physical stability compared to the neat amorphous components when investigated by an independent experimental method; and second, to identify the sequence and nature of phase formation during crystallization of this coamorphous mixture.

The time-resolved XRD patterns collected at 363 K for neat ITR, neat POS, and the coamorphous eutectic mixture are presented in Figure 8. In the case of neat substances, both ITR and POS undergo rapid recrystallization, with distinct, sharp diffraction peaks appearing within 90–180 min, indicating limited physical stability under the applied conditions. In contrast, the eutectic coamorphous mixture remains X-ray amorphous for a significantly longer period, with no clear signs of recrystallization until approximately 6.5–23 h of storage, suggesting delayed crystallization. This pronounced delay in the onset of crystallization confirms the superior physical stability of the coamorphous system at eutectic concentration, in agreement with the crystallization kinetic data obtained by BDS. Notably, a direct comparison of the PXRD patterns (see Figure 9) reveals that the most intense diffraction peaks observed after crystallization of the ITR + 51.4 wt % POS mixture do not match the characteristic reflections of either pure ITR or pure POS, which are marked by blue and green dashed arrows, respectively. Specifically, the three strongest peaks of each pure component are absent in the recrystallized POS + 51.4 wt % POS sample. Instead, the strongest five dominant reflections appear (highlighted by red-shaded regions in Figure 9), partially overlapping with signals from both individual components, yet not fully attributable to either. This observation suggests the formation of a crystalline phase distinct from those of the parent ITR and POS compounds. Based solely on the presented diffraction data, however, its structural nature cannot be unambiguously determined. The observed diffraction pattern may result from cocrystallization, the formation of polymorphic forms of neat components but different from those of the parent compounds, or another type of crystalline reorganization induced by mixing.^32^

**Figure 8:**
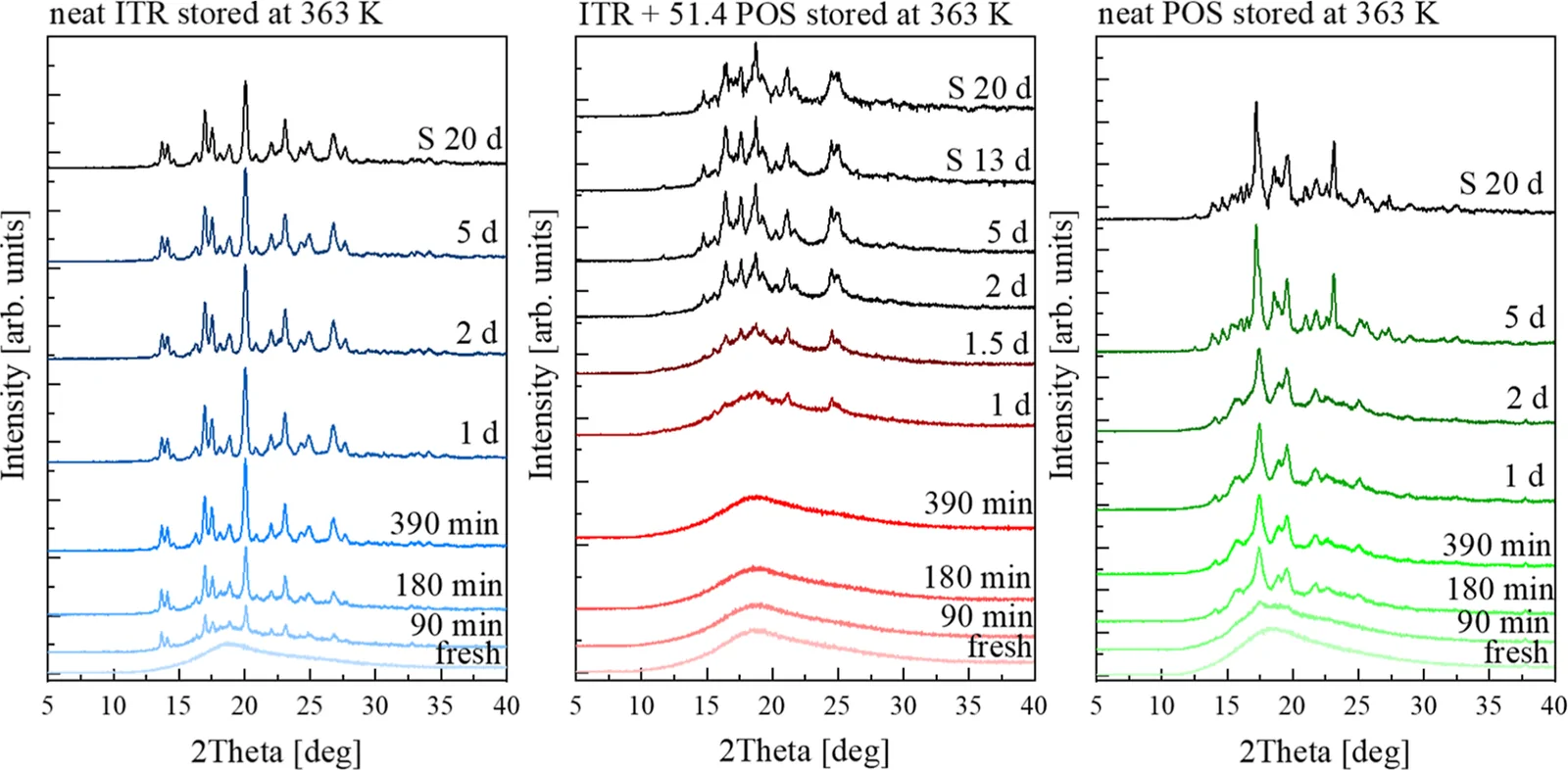
PXRD patterns of neat ITR, neat POS, and the eutectic coamorphous mixture (ITR + 51.4 wt % POS) after storage at 363 K for 90, 180, 390 min, and 1, 2, 5, and 20 days. In each panel, bottom diffractogram represents the freshly prepared amorphous sample (0 min).

**Figure 9:**
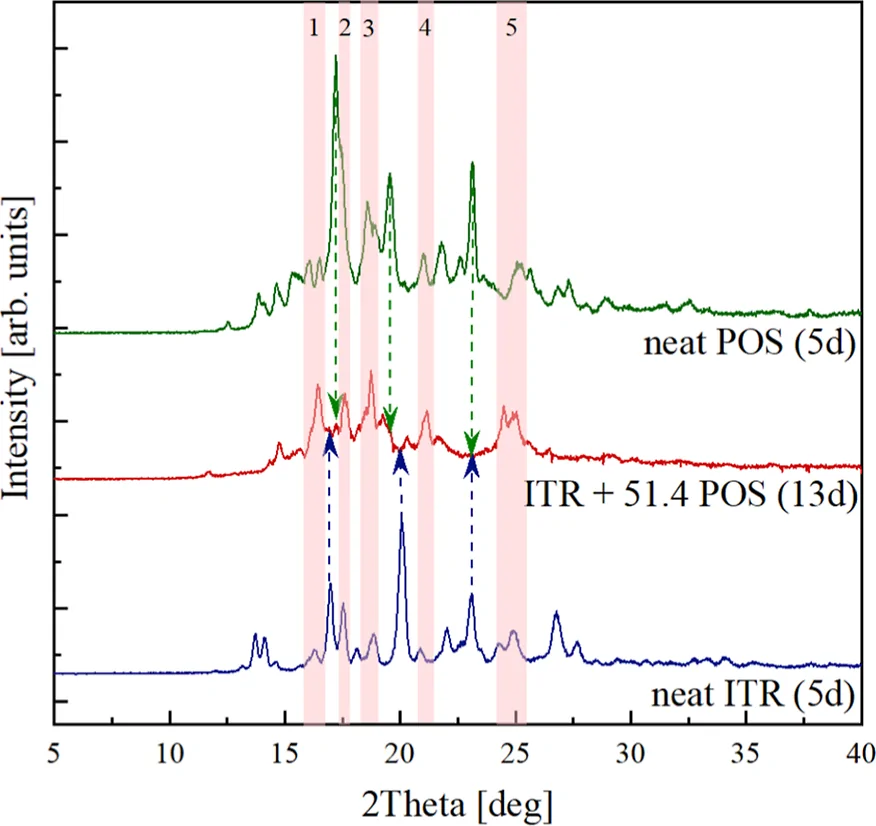
XRD patterns of neat POS (5 days at 363 K), neat ITR (5 days at 363 K), and the coamorphous eutectic mixture ITR + 51.4 wt % POS (13 days at 363 K). The most intense peaks of neat POS and ITR are marked with green and blue arrows, respectively. Shaded regions (1–5) indicate the dominant reflections observed in the crystallized eutectic mixture.

### 3.5 Physical Stability Studies of Glassy ITR + POS Systems

To evaluate the long-term physical stability of glassy neat ITR, neat POS, and their coamorphous eutectic mixture (ITR + 51.4 wt % POS), XRD measurements were conducted after storage under standard pharmaceutical conditions (i.e., *T* = 298 K, *RH* = 60%) for up to 35 days. The samples, initially prepared in the fully amorphous state, were investigated at selected time points to monitor potential recrystallization. Figure 10 shows the obtained XRD patterns of the amorphous samples at different storage intervals (1, 7, 14, and 35 days), along with the corresponding patterns of fully crystalline references (crystalline ITR, crystalline POS, and their physical mixture). The diffraction pattern of the crystalline physical mixture was additionally compared with the calculated weighted sum of the diffraction patterns of neat crystalline ITR and POS at the eutectic composition. The good agreement between these two patterns suggests that the crystalline physical mixture represents a simple superposition of the parent crystalline phases. As can be seen for all three materials, the absence of sharp Bragg peaks and the persistence of a broad halo characteristic of amorphous systems indicate that no detectable recrystallization occurred during the 35-day storage period.^33–35^ This behavior contrasts with the faster recrystallization observed at elevated temperatures (as shown previously in Figures 8 and 9), highlighting the critical role of storage conditions in maintaining the amorphous state. Moreover, it underlines the importance of accelerated physical stability studies conducted under stress conditions, such as increased temperature, to effectively assess the stabilization potential of coamorphous systems and to identify formulations with enhanced resistance to devitrification.

**Figure 10:**
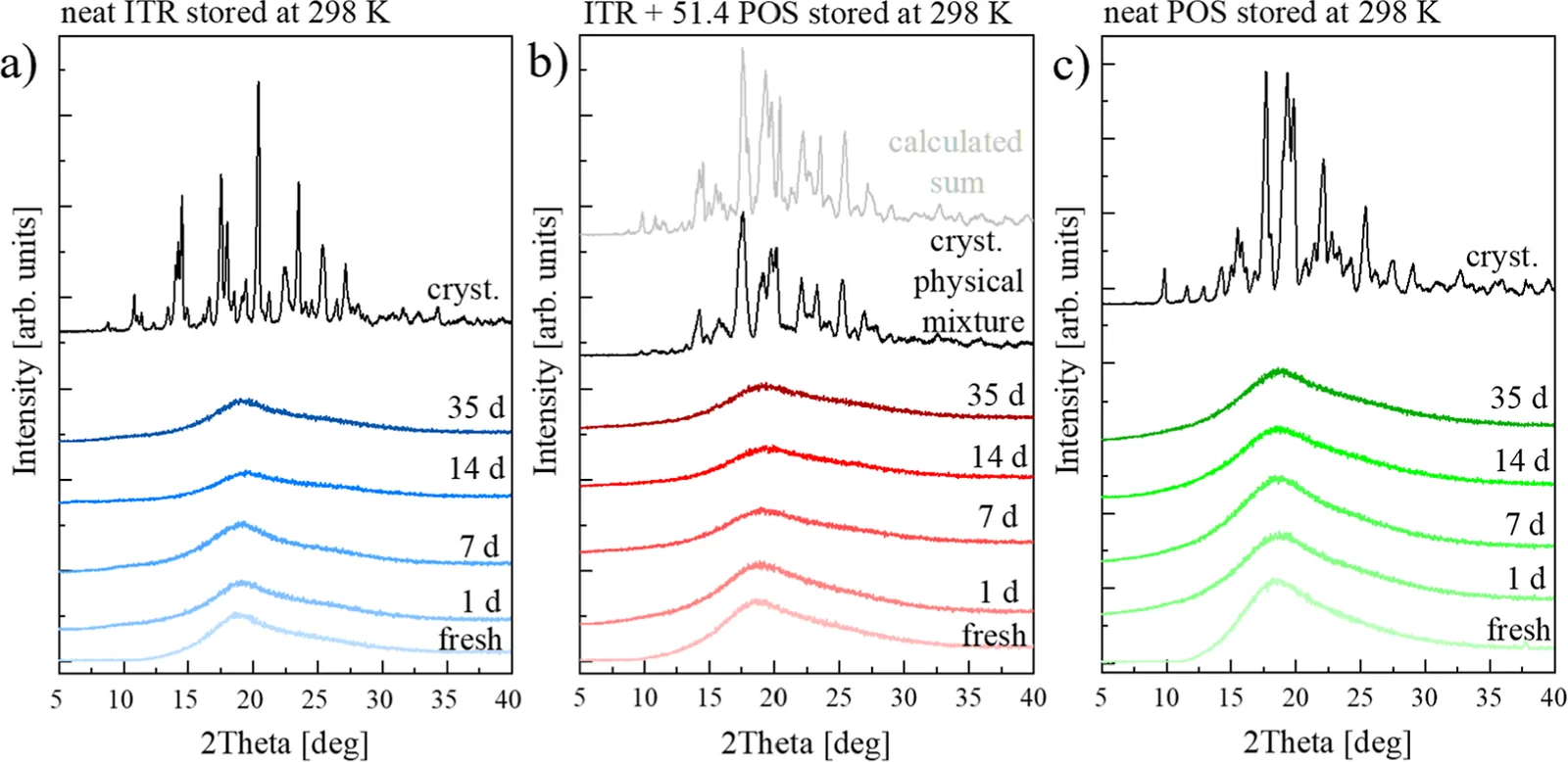
PXRD patterns of neat ITR, neat POS, and the eutectic coamorphous mixture (ITR + 51.4 wt % POS) after storage at 298 K and 60% RH for 1, 7, 14, and 35 days. In each panel, the black diffractogram corresponds to the fully crystalline reference (either the neat crystalline form or the physical mixture of crystalline components), while the bottom diffractogram represents the freshly prepared amorphous sample (day 0).

## 4 Conclusions

In this work, we systematically investigated the thermal behavior of crystalline and amorphous mixtures of itraconazole (ITR) and posaconazole (POS) across the entire compositional range. Through differential scanning calorimetry (DSC), we identified a eutectic composition at 51.4 wt % POS and demonstrated that the ITR + POS binary system follows nearly ideal mixing behavior in the molten state. The quench-cooled mixtures exhibited similar glass transition temperatures (*T*_*g*_), ranging from 332 to 334 K at a heating rate of 10 K/min. Remarkably, for the coamorphous mixture at the eutectic composition, a complete suppression of the liquid crystalline (LC) ordering of ITR was observed. This phenomenon, not previously reported for amorphous pharmaceutical systems, might suggest a unique ability of eutectics to disrupt LC alignment of one of the mixture components. Dielectric studies confirmed this behavior: the ITR + POS system at the eutectic composition exhibited neither δ-relaxation features nor LC-associated transitions, indicating a fully disordered amorphous structure. These experiments also revealed subtle changes in molecular dynamics, including a slight broadening of the α-relaxation process and an increase in the *T*_*g*_ value with increasing POS content.

Isothermal dielectric studies confirmed a significant enhancement in physical stability at the eutectic composition, which exhibited the longest induction times, the slowest crystallization kinetics, and the highest Avrami exponent among all tested formulations. Complementary X-ray diffraction (XRD) analyses confirmed these findings and additionally revealed the formation of a crystalline phase distinct from those observed for the parent compounds upon devitrification of the eutectic coamorphous system. The exact structural nature of this phase remains to be established and may involve different forms of crystalline reorganization, including cocrystallization or polymorphic transformation.

Taken together, our findings demonstrate that the eutectic ITR + POS mixture in the amorphous state benefits mutually from both components: on the one hand, the suppression of LC ordering in ITR is expected to improve its solubility, while on the other hand, enhanced molecular interactions help stabilize the inherently physically unstable amorphous POS. The synergistic improvement achieved through coamorphization of the ITR + POS system offers a promising formulation strategy for drugs that are poorly soluble in the crystalline state and physically unstable in the amorphous form.

## References

[1] Thakkar H. P., Khunt A., Dhande R. D., Patel A. A. (2015). Formulation and
evaluation of Itraconazole nanoemulsion for enhanced oral bioavailability. J. Microencapsul..

[2] Vuletić L., Herceg M., Ferderber K., Tunjić I., Rizea-Savu S., Duna S. N., Cetina-Čižmek B., Filipović-Grčić J. (2019). Single-Dose Pharmacokinetic Properties
and Relative Bioavailability of Different Formulations of Posaconazole
Oral Suspension in Healthy Volunteers. Clin.
Pharmacol. Drug Dev..

[3] Marano S., Ghimire M., Missaghi S., Rajabi-Siahboomi A., Craig D. Q. M., Barker S. A. (2023). Development of Robust Tablet Formulations
with Enhanced Drug Dissolution Profiles from Centrifugally-Spun Micro-Fibrous
Solid Dispersions of Itraconazole, a BCS Class II Drug. Pharmaceutics.

[4] Hens B. (2018). Evaluation and optimized
selection of supersaturating drug delivery
systems of posaconazole (BCS class 2b) in the gastrointestinal simulator
(GIS): An in vitro-in silico-in vivo approach. Eur. J. Pharm. Sci..

[5] Tao T., Zhao Y., Wu J., Zhou B. (2009). Preparation and evaluation
of itraconazole dihydrochloride for the solubility and dissolution
rate enhancement. Int. J. Pharm..

[6] Fang, L. Y. ; Wan, J. ; Harris, D. Oral Pharmaceutical Compositions in a Solid Dispersion Comprising Preferably Posaconazole and HPMCAs. U.S. Patent 20,110,034,478 A1, 2011.

[7] Zhang S., Lee T. W. Y., Chow A. H. L. (2016). Crystallization
of Itraconazole Polymorphs
from Melt. Cryst. Growth Des..

[8] Tarnacka M. (2013). Molecular dynamics of
itraconazole at ambient and high pressure. Phys.
Chem. Chem. Phys..

[9] Six K. (2001). Investigation of thermal properties of glassy itraconazole: identification
of a monotropic mesophase. Thermochim. Acta.

[10] Nijhawan M., Godugu M., Saxena T., Farheen T., Dwivedi K. (2022). Pharmaceutical
co-crystals of posaconazole for improvement of physicochemical properties. Braz. J. Pharm. Sci..

[11] Fule R., Amin P. (2014). Hot Melt Extruded Amorphous
Solid Dispersion of Posaconazole with
Improved Bioavailability: Investigating Drug-Polymer Miscibility with
Advanced Characterisation. BioMed Res. Int..

[12] Baird J. A., Van Eerdenbrugh B., Taylor L. S. (2010). A Classification
System to Assess
the Crystallization Tendency of Organic Molecules from Undercooled
Melts. J. Pharm. Sci..

[13] Kramarczyk D., Knapik-Kowalczuk J., Kurek M., Jamróz W., Jachowicz R., Paluch M. (2023). Hot Melt Extruded Posaconazole-Based
Amorphous Solid Dispersions—The Effect of Different Types of
Polymers. Pharmaceutics.

[14] Adrjanowicz K. (2013). Molecular Dynamics of
the Supercooled Pharmaceutical Agent Posaconazole
Studied via Differential Scanning Calorimetry and Dielectric and Mechanical
Spectroscopies. Mol. Pharmaceutics.

[15] Yang F., Su Y., Brown C. D., DiNunzio J. (2021). Solubilizing temperature of crystalline
drug in polymer carrier: A rheological investigation on a posaconazole-copovidone
system with low drug load. Eur. J. Pharm. Biopharm..

[16] Guidetti M., Hilfiker R., Kuentz M., Bauer-Brandl A., Blatter F. (2024). Water-mediated phase transformations
of posaconazole:
An intricate jungle of crystal forms. Eur. J.
Pharm. Sci..

[17] Herman C., Haut B., Aerts L., Leyssens T. (2012). Solid–liquid
phase diagrams for the determination of the solid state nature of
both polymorphs of (RS)-2-(2-oxo-pyrrolidin-1-yl)-butyramide. Int. J. Pharm..

[18] Amponsah-Efah K. K., Glorieux C., Thoen J., Suryanarayanan R. (2020). Effect of
glycerol on the order of the mesophase transitions of supercooled
itraconazole. J. Mol. Liq..

[19] Mapesa E. U. (2014). Molecular dynamics of itraconazole confined in thin supported layers. RSC Adv..

[20] Kolodziejczyk K., Paluch M., Grzybowska K., Grzybowski A., Wojnarowska Z., Hawelek L., Ziolo J. D. (2013). Relaxation dynamics
and crystallization study of sildenafil in the liquid and glassy states. Mol. Pharmaceutics.

[21] Schönhals, A. ; Kremer, F. Analysis of Dielectric Spectra. In Broadband Dielectric Spectroscopy 2003, pp 59–98. Springer. Berlin, Heidelberg. 10.1007/978-3-642-56120-7_3 .

[22] Yamamuro O. (1999). Glass Transitions
of Molecular Materials Studied by Calorimetry - Focused on High Pressure
Experiments. Rev. High Press. Sci. Technol..

[23] Fulcher G. S. (1925). Analysis
of recent measurements of the viscosity of glasses. J. Am. Ceram. Soc..

[24] Tammann G., Hesse W. (1926). The dependence of viscosity
on the temperature of supercooled liquids. J.
Inorganic General Chem..

[25] Kissi E. O., Khorami K., Rades T. (2019). Determination of Stable
Co-Amorphous
Drug–Drug Ratios from the Eutectic Behavior of Crystalline
Physical Mixtures. Pharmaceutics.

[26] Knapik-Kowalczuk J., Kramarczyk D., Jurkiewicz K., Chmiel K., Paluch M. (2021). Ternary Eutectic
Ezetimibe–Simvastatin–Fenofibrate System and the Physical
Stability of Its Amorphous Form. Mol. Pharmaceutics.

[27] Noor W., Macovez R., Zamora P. M., Tamarit J. L., Romanini M. (2024). Eutectic binary
phase diagram and kinetically stable homogeneous co-amorphous mixtures
of two imidazole drugs. J. Mol. Liquids.

[28] Cichocka-Łokuciejewska J., Knapik-Kowalczuk J., Dulski M., Greber K. E., Sawicki W., Paluch M. (2026). Eutectic Coamorphous
System of Enzalutamide and Acetyl
Maltose: A Strategy for Improved Physical Stability and Aqueous Solubility. Mol. Pharmaceutics.

[29] Poloczek P., Knapik-Kowalczuk J., Klimontko J., Han X., Kawakami K., Paluch M. (2026). Eutectic-Driven
Recrystallization of Coamorphous Bicalutamide
+ Niclosamide Systems: Contrasting Stability above and below Tg. Mol. Pharmaceutics.

[30] Noor W., Romanini M., Villalobos L., Talukder B., Tamarit J. L., Macovez R. (2026). Metastable phase diagram,
mobility, and kinetic stability
of amorphous mixtures of two mutually compatible APIs. Int. J. Pharm.:X.

[31] Grzybowska K., Capaccioli S., Paluch M. (2016). Recent developments in the experimental
investigations of relaxations in pharmaceuticals by dielectric techniques
at ambient and elevated pressure. Adv. Drug
Deliv. Rev..

[32] Berry D. J., Seaton C. C., Clegg W., Harrington R. W., Coles S. J., Horton P. N., Hursthouse M. B., Storey R., Jones W., Friščić T. (2008). Applying hot-stage microscopy to co-crystal screening: A study of
nicotinamide with seven active pharmaceutical ingredients. Cryst. Growth Des..

[33] Newman A. W., Byrn S. R. (2003). Solid-state analysis of the active pharmaceutical ingredient
in drug products. Drug Discov. Today.

[34] Wu W., Ueda H., Löbmann K., Rades T., Grohganz H. (2018). Organic acids
as co-formers for co-amorphous systems – Influence of variation
in molar ratio on the physicochemical properties of the co-amorphous
systems. Eur. J. Pharm. Biopharm..

[35] Hancock B. C., Zografi G. (1997). Characteristics and Significance
of the Amorphous State
in Pharmaceutical Systems. J. Pharm. Sci..

